# Editorial: EEG-based assistive robotics for rehabilitation

**DOI:** 10.3389/fnbot.2022.952495

**Published:** 2022-07-14

**Authors:** Wajid Mumtaz, Hafeez Ullah Amin, Abdul Qayyum, Ahmad Rauf Subhani

**Affiliations:** ^1^Department of Electrical Engineering, School of Electrical Engineering and Computer Science, National University of Sciences and Technology, Islamabad, Pakistan; ^2^School of Computer Science, University of Nottingham Malaysia, Semenyih, Malaysia; ^3^Department Vision and Robotics, Université de Bourgogne, Dijon, France; ^4^Department of Electrical Engineering, College of Electrical and Mechanical Engineering, National University of Sciences and Technology, Islamabad, Pakistan

**Keywords:** EEG, motor disability, BCI, robotics, rehabilitation

Electroencephalography (EEG) is a well-known neuroimaging modality, having benefits such as low cost and high time-resolution. It has been shown tremendous potential in diagnosing mental health issues such as learning disability, cognitive therapy like neurofeedback, and rehabilitation systems, such as brain-computer interfaces (BCI) (Malik and Amin, 2017). EEG-based BCI systems have been developed into assistive robots, such as BCI robotic systems for stroke (Ang et al., 2015), BCI systems for a wheelchair (Tang et al., 2018), and BCI robots for hand movement (Ofner et al., 2019).

Furthermore, the utility of data processing with deep learning architectures is becoming more prominent. With the advancements in the processing capability of the underlying hardware resources, real-time applications can be developed. However, potential challenges are there in developing EEG-based assistive robotic systems, for instance, real-time EEG analysis, discriminating neural events concerning the physical events, and efficiency in communicating the EEG from the brain to devices. In addition, the challenge of low signal-to-noise ratio (SNR) inherent in EEG data can be addressed at multiple levels, such as during the recording and pre-processing stages, where a bunch of sophisticated techniques can be used for the automatic removal of artifacts at runtime.

Addressing such potential challenges demands new research methods to enhance the EEG-based robots for rehabilitation. For instance, convolutional neural networks (CNNs) allow the automatic adjustment of the input kernels for extracting the most relevant features that could help the classification at the later stages (Liu and Yang, 2021). This way, the low SNR challenge can be addressed as well. Therefore, this particular Research Topic in Frontiers in Robotics and AI aims to cover recent developments in robots for rehabilitation based on EEG technology and bring the latest research articles embarking on the cutting-edge technology for biomedical devices and applications that help in BCIs. Under this Research Topic, four articles were finally accepted for publication, addressing some of the potential challenges, like improvement in SNR. A brief overview is provided on four published articles as follows.

Kumar et al. have proposed a novel brain-computer interface (BCI) based robot-assisted stroke rehabilitation system (RASRS), which takes inputs from the patient's intrinsic feedback mechanism to adapt the assistance level of the RASRS. The proposed system will utilize the patients' consciousness about their performance decoded through their error-related negativity signals. As a proof-of-concept, a sample of 12 healthy people was used in experimentation in which their EEG signals were captured while performing a standard rehabilitation exercise. The authors reported a distinct neural response of participants when failing to do a rehabilitation exercise compared to when they succeeded in a specified time. In addition, neural activity detected in a single trial from background EEG activity is essential for developing a rehabilitation system.

Accurate decoding of motor imagery (MI)-EEG signals is essential for effective BCI systems. However, the presence of noise in EEG signals makes it difficult. Thus, Yang et al. propose a deep learning decoding method based on multi-hierarchical representation fusion (MHRF) on MI-EEG. The proposed method consists of a concurrent framework constructed of bidirectional LSTM (Bi-LSTM) and convolutional neural network (CNN) to fully capture the contextual correlations of MI-EEG and the spectral feature. Also, the stacked sparse autoencoder (SSAE) is employed to concentrate these two domain features into a high-level representation for cross-session and subject training guidance. The authors have reported the efficacy and practicality of the proposed method by testing it on a public dataset from BCI competition IV as well as a collected in performing MI task. It is also stated that the proposed method has the potential to improve inter-session and inter-subject transferability, essential for the practical implementation of a calibration-free BCI system.

Mental stress can degrade the cognitive performance of an individual due to external burdens. Al-Saggaf et al. investigates the performance of computer-aided EEG-based approaches for mental stress assessment for Rehabilitation. The authors compared the Machine learning (ML) approaches to the time required for feature extraction and classification. It was observed after using a real-time experiment that conventional ML could be time-consuming due to computations cost for feature extraction as compared to deep ML, because of its automated unsupervised feature extraction. The authors recommend that deep machine learning could be used in wearable devices for real-time EEG-based mental stress assessment for rehabilitation.

Individuals with motor disabilities demand to improve or replace their movement functions to interact with objects. Xu et al. have investigated decoding five different reach-and-grasp movements closely related to daily life using movement-related cortical potentials (MRCPs). A nine healthy subjects' sample is exposed to an experiment in which the subjects were asked to execute five different reach-and-grasp movements, including palmar, pinch, push, twist, and plug grasp. Each subject performed 80 trials per condition, resulting in a total of 480 trials being captured. The study used EEG low-frequency range for feature extraction for decoding purposes and reported promising classification results for the five experimental conditions. The authors showed that in the MRCPs all the grasping conditions were more pronounced than the no-movement condition. The findings could be useful in natural and intuitive BCI applications, including rehabilitation robots.

## Author contributions

All authors contributed to the editorial drafting, read, and approved. All authors contributed to the article and approved the submitted version.

## Conflict of interest

The authors declare that the research was conducted in the absence of any commercial or financial relationships that could be construed as a potential conflict of interest.

## Publisher's note

All claims expressed in this article are solely those of the authors and do not necessarily represent those of their affiliated organizations, or those of the publisher, the editors and the reviewers. Any product that may be evaluated in this article, or claim that may be made by its manufacturer, is not guaranteed or endorsed by the publisher.
